# Detection of *Mycobacterium tuberculosis* complex DNA in oronasal swabs from infected African buffaloes (*Syncerus caffer*)

**DOI:** 10.1038/s41598-022-05982-6

**Published:** 2022-02-03

**Authors:** Charlene Clarke, David V. Cooper, Michele A. Miller, Wynand J. Goosen

**Affiliations:** 1 DSI-NRF Centre of Excellence for Biomedical Tuberculosis Research, South African Medical Research Council Centre for Tuberculosis Research, Division of Molecular Biology and Human Genetics, Faculty of Medicine and Health Sciences, Stellenbosch University, PO Box 241, Cape Town, 8000 South Africa; 2 Ezemvelo KwaZulu-Natal Wildlife, PO Box 25, Mtubatuba, 3935 South Africa

**Keywords:** Tuberculosis, Translational research

## Abstract

*Mycobacterium bovis* (*M. bovis*), a member of the *Mycobacterium tuberculosis* complex (MTBC), is the causative agent of bovine TB (bTB) in animals. Spread occurs through inhalation or ingestion of bacilli transmitted from infected individuals. Early and accurate detection of infected African buffaloes shedding *M. bovis* is essential for interrupting transmission. In this pilot study, we determined if MTBC DNA could be detected in *M. bovis* infected buffalo oronasal secretions using a molecular transport media (PrimeStore MTM) with oronasal swabs and a rapid qPCR assay (Xpert MTB/RIF Ultra). Bovine TB test-positive buffaloes were culled, then tissue samples and oronasal swabs collected post-mortem for mycobacterial culture and Ultra testing, respectively. The Ultra detected MTBC DNA in 5/12 swabs from *M. bovis* culture-confirmed buffaloes. Oronasal swabs from *M. bovis* negative buffaloes (n = 20) were negative on Ultra, indicating the high specificity of this test. This study showed that MTM can successfully preserve MTBC DNA in oronasal swabs. The proportion of MTBC positive oronasal swabs was higher than expected and suggests that the Ultra may be an additional method for identifying infected buffaloes. Further studies are needed to confirm the utility of the Ultra assay with oronasal swabs as an assay to evaluate possible MTBC shedding in buffaloes.

## Introduction

Bovine tuberculosis (bTB) is a debilitating chronic infectious disease in livestock and wildlife species, caused by the slow-growing aerobic bacteria of the *Mycobacterium tuberculosis* complex (MTBC), primarily *Mycobacterium bovis*^1^. Bovine TB may have devastating economic, social, and environmental consequences for animal populations and communities that are dependent on production animals, wildlife trade, tourism, and conservation programs. Although bTB is actively managed through control programs, such as test-and-slaughter, complete elimination of disease is complicated by the presence of maintenance hosts in both livestock and wildlife environments^2,3^. In South Africa, African buffaloes (*Syncerus caffer*) are important wildlife maintenance hosts of *M. bovis* and responsible for persistent infection in wildlife populations through spill-overs between buffaloes and other wildlife species, as well as cattle bordering wildlife reserves^4,5^.

Shedding of mycobacteria primarily occurs through the release of viable bacilli in airborne particles from the host’s pulmonary or laryngeal lymph node TB lesions^6,7^. Transmission may also occur with inhalation of particles in faecal and urinary excretions originating from extrapulmonary lesions^6,7^. Mycobacterial bacilli are small, nonmotile rods with a waxy mycolic acid cell wall that allows them to survive in the environment outside the host up to several months, depending on environmental conditions^1,6^. This allows infection of hosts indirectly through ingestion or inhalation of bacilli shed from infected individuals into the environment. Buffaloes are effective maintenance hosts of bTB due to their gregarious nature, with close social contact and susceptibility leading to spread within a herd^4^. Buffaloes may harbour *M. bovis* for months to years, remaining a source of infection to predators and other animals sharing its environment^4,5^.

Interruption of *M. bovis* transmission is central to achieving reduction in bTB incidence; therefore, early detection of infected animals, especially those shedding, is essential to prevent further transmission of the pathogen. Diagnosis of bTB typically relies on the measurements of delayed-type hypersensitivity responses to the intradermal injection of tuberculin, known as the tuberculin skin test (TST), and in vitro antigen-specific cell-mediated immune responses. These are typically based on cytokine release in plasma from whole blood stimulated with the TB antigens, early secretory antigenic target protein 6 (ESAT-6) and culture filtrate protein 10 (CFP-10), or bovine and avian tuberculins. The bovine interferon-gamma (IFN-γ) release assay (IGRA) and IFN-γ-induced protein 10 (IP-10) assay (IPRA) have been adapted from assays developed for cattle for use in buffaloes^2,8^. However, confirmation of *M. bovis* infection requires direct detection of the organism, which is usually based on post-mortem mycobacterial culture and genetic speciation of mycobacterial isolates from collected tissue samples^9^; however, conventional mycobacterial culture methods are time-consuming, taking up to 56 days before results are available, and have limited sensitivity^2,10^. Therefore, other more rapid direct methods are needed to detect the presence of bacilli, particularly ante-mortem, to confirm infection and evaluate mycobacterial transmission risk to other animals.

Oronasal swab samples for identification of MTBC DNA using an automated rapid qPCR, the Xpert MTB/RIF Ultra (Ultra) assay (Cepheid, Sunnyvale, California, USA) have been shown to be an effective, alternative to sputum collection for *M. tuberculosis* diagnosis in humans, especially in patients with other underlying conditions, such as HIV, and in children that are unable to produce these samples^11,12^. Sputum collection in animals is not feasible for bTB diagnosis and collection of other airway secretions, for example, bronchoalveolar lavage, are invasive and time-consuming. Oronasal swabs may provide a more easily obtained alternative source for detection of MTBC. A previous study has shown that the Ultra assay could effectively identify MTBC DNA in swabs of buffalo tissue samples collected post-mortem, with results available in less than two hours^13^. Since lesions in infected animals are typically found in the respiratory system, mycobacteria in droplets, that pass through the oronasal cavities and adhere to epithelia, or are released from lesions in the oropharyngeal lymph nodes or lung, could potentially be detected by the Ultra assay^11^. Therefore, detection of MTBC DNA by Ultra in these samples may provide evidence of infection^12^. Furthermore, collection of oronasal swabs is an easy, inexpensive, rapid method of sample collection and, together with Ultra, could reveal shedding in buffaloes, which would be a tool to evaluate transmission risk for epidemiological studies. Therefore, the aim of this pilot study was to investigate the application of the Ultra assay, combined with the oronasal swab samples collected from *M. bovis* culture-confirmed infected and uninfected buffaloes, stored in a commercially available nucleic acid stabilising, pathogen inactivating transport media (PrimeStore MTM) to detect MTBC DNA. This could then be further evaluated as a useful ante-mortem screening test for *M. bovis* shedding in African buffaloes.

## Results

Oropharyngeal swabs were collected from 15 Hluhluwe–iMfolozi Park (HiP) buffaloes in PrimeStore molecular transport medium (MTM) during post-mortem examinations. Of these, 12 buffaloes were confirmed *M. bovis* infected, based on mycobacterial culture of tissues and downstream genotyping of isolates by region of difference (RD) polymerase chain reaction (PCR)^14^; the remaining three buffaloes were negative on all bTB tests (IPRA, IGRA and single comparative intradermal tuberculin test, SCITT) and mycobacterial tissue culture (Table 1). The Ultra assay detected MTBC DNA in 5 of 12 (42%) PrimeStore MTM oronasal swab samples from culture-positive animals; notably, *M. bovis* was isolated from the head and thoracic lymph node tissues from four of five (80%) of these buffaloes (Table 1). All three (100%) *M. bovis* culture-negative HiP buffaloes had a negative Ultra result from the swabs.

**Table 1:** Mycobacterial culture, Xpert MTB/RIF Ultra assay, and *Mycobacterium tuberculosis* complex antigen-specific immunological tests (IP-10 release assay, interferon-gamma release assay, single comparative intradermal tuberculin test) results for HiP buffaloes. Shaded blocks indicate a positive test result.

Buffalo ID	Tissue samples		Oropharyngeal swabs	Bovine TB immunological assays		
	Tissue samples collected for mycobacterial culture	*M. bovis* culture and RD test result	Xpert MTB/RIF Ultra test result	IGRA (S/P%)	IPRA (pg/ml)	∆ SCITT
A107	L&R Retropharyngeal LN	Positive	MTBC not detected	**49**	0	**28.5**
A113	Tonsils	Positive	MTBC not detected	**26**	**3487**	**30.2**
A20	Tonsils	Positive	**MTBC trace detected**	**29**	**5228**	**6.7**
A98	Tonsils & retropharyngeal LN	Positive	**MTBC detected very low**	**55**	**5591**	**28.7**
B15	Lung lesion	Positive	MTBC not detected	**22**	**5046**	0.7
B19	Retropharyngeal LN	Positive	**MTBC trace detected**	**60**	**1773**	**29.4**
B30	Mediastinal LN lesions	Positive	MTBC not detected	2	**1675**	**9.3**
B48	R Retropharyngeal LN lesion	Positive	MTBC not detected	**60**	**9479**	**3.1**
B64	Abdominal serosa	Positive	**MTBC detected very low**	**94**	663	0.1
B65	L&R Tracheobronchial LN lesions	Positive	MTBC not detected	**225**	**3224**	**29.3**
B8	L&R Tracheobronchial LN lesions	Positive	MTBC not detected	**60**	**2263**	**29.5**
C28	Tonsil lesion; retropharyngeal LN	Positive	**MTBC detected very low**	**46**	1049	**9.1**
A145	Mediastinal LN	Negative	MTBC not detected	1	10	−0.9
A149	Lung	Negative	MTBC not detected	0.96	319.9	0.9
B62	Lung lesion	Negative	MTBC not detected	−5.2	661.6	2.2

IGRA = IFN-γ release assay; IPRA = IP-10 release assay; SCITT = single comparative intradermal tuberculin test; L = left; R = right; LN = lymph node; MTBC = *M. tuberculosis* complex*;* RD = region of difference.

The high specificity of the Ultra assay was confirmed by the negative results for oronasal swab samples collected from buffaloes in historically bTB negative game parks (n = 20; data not shown).

Since positive Ultra results could have been due to oronasal contamination with MTBC from the environment or other herd members, additional ante-mortem test results were evaluated. All *M. bovis* infected buffaloes (n = 12) had at least one positive result among the three immunological assays, i.e., SCITT, IPRA and IGRA, which were used to screen these animals before culling.

## Discussion

The aim of this pilot study was to determine whether MTBC DNA could be detected in *M. bovis* infected buffalo, by using oronasal swabs, stored in a commercially available nucleic acid stabilising, pathogen inactivating transport media (PrimeStore MTM) with the rapid Xpert MTB/RIF Ultra assay. In a cohort of 12 culture-confirmed *M. bovis* infected buffaloes, each with at least one positive antigen-specific immunological test result (SCITT, IPRA, IGRA), 5 individuals had an Ultra positive oronasal swab result, which supports the hypothesis that these were infected animals possibly shedding mycobacteria. Although environmental contamination of oronasal secretions with MTBC could not definitively be ruled out, the isolation of *M. bovis* from head and thoracic lymph nodes from four of five Ultra positive buffaloes, along with a documented immunological response, suggests that the animals were truly infected and the source of the MTBC DNA. Moreover, the three *M. bovis* exposed (although culture and immunological test negative) buffaloes shared the same environment as the rest of the HiP cohort, and were culled and sampled under the same circumstances, but had negative Ultra results, indicating that the detection of MTBC DNA in the five Ultra positive buffaloes were not due to environmental contamination.

The results in this study further demonstrated that the method of collection and inactivation using PrimeStore MTM to store oronasal swab samples did not interfere with detection of MTBC DNA using the Ultra assay. A previous study assessed the use of PrimeStore MTM with swabs collected from buffalo tissue samples to detect MTBC DNA by Ultra; this was found to be a safe, easy, and effective method to transport samples at room temperature to screen animals for the presence of *M. bovis*^13^. The current study has shown that PrimeStore MTM can also be used with oronasal swabs that, when used with the Ultra assay, provides a simple method for detecting MTBC DNA in oronasal secretions, making it a potential tool for evaluating shedding.

All buffaloes (excluding the three buffaloes that were culture and immunological test negative) were culled, based on their positive antigen-specific immunological test results (SCITT, IGRA and/or IPRA), which provided an indirect indication of infection. Twelve buffaloes were confirmed to be *M. bovis* infected by mycobacterial culture from tissues. However, other studies suggest that culture is an imperfect method for detection of *M. bovis* infection^2,10^. Conventional mycobacterial culture uses a relatively harsh decontamination treatment and samples may be subjected to freeze–thaw cycles prior to culture, both of which may reduce the viability of mycobacteria that may be present in small numbers in oropharyngeal swabs^15^. Mycobacterial culture also has poor sensitivity and takes up to 56 days for adequate growth; therefore, culture of swabs may be a poor tool to assess shedding in buffalo herds. However, use of a highly sensitive qPCR assay (Ultra), in combination with samples stored in PrimeStore MTM, which stabilises the nucleic acids in the swab samples, may facilitate positive identification of paucibacillary MTBC DNA in oronasal secretions, which would increase the proportion of test-positive infected buffaloes.

Four of five buffaloes, in which possible *M. bovis* shedding in oropharyngeal secretions was detected by Ultra, had *M. bovis* culture-positive results from head and thoracic lymph nodes. One animal (B64), in which possible MTBC DNA shedding was also detected, had generalised *M. bovis* culture-positive lesions in the abdominal serosa, indicative of extrapulmonary bTB. Studies have found that the distribution and progression of lesion development are correlated to the route of mycobacterial infection^16^. For example, cattle, infected endotracheally with *M. bovis,* had culture-positive gross pathological lesions in the lungs and thoracic cavity, whereas those that were orally infected had lesions in abdominal tissues^16^. Gastrointestinal lesions may also be caused by swallowing infectious respiratory secretions or through lymphatic spread^17,18^. Although only a single case, the Ultra result from buffalo B64 suggests that buffaloes with extrapulmonary bTB may also be capable of shedding. Moreover, even though the anatomical location of lesions provides some information on possible routes and sites of infection, it cannot be used to definitively determine whether the individual is infectious and shedding. For example, *M. bovis* was cultured from faeces in cattle that only had bTB lesions in thoracic tissues^17^. Therefore, further studies on the pathways of *M. bovis* excretion in buffaloes are required.

One of the limitations of the study is that it could not be definitively determined if detection of MTBC DNA in oronasal swabs was a result of shedding or environmental contamination. Positive antigen-specific immunological test results in Ultra assay positive buffaloes provide further evidence that positive Ultra results were found in truly infected buffaloes. In addition, the high specificity of the Ultra assay was supported by the negative results from historically bTB negative buffalo herds, which were in environments that may contain environmental mycobacteria. Therefore, these results indicate that oronasal swabs with a positive Ultra result were detecting MTBC DNA, rather than nontuberculous mycobacteria. However, future studies should include oronasal swabs from larger cohorts of *M. bovis* uninfected buffaloes in bTB endemic environments to determine if the Ultra assay can detect oronasal contamination with MTBC.

An additional limitation was that since the swabs were stored in PrimeStore MTM, which inactivates microorganisms, the swabs could not be cultured and therefore direct verification of the Ultra results by determining the presence of viable bacilli could not be performed. Finally, only a single swab was collected from each buffalo, which represents a single time point. If the buffaloes were serially sampled over time, more animals may have been detected as potentially shedding in this cohort of infected buffaloes.

In summary, this pilot study supports the use of the Ultra assay with oronasal swabs to screen for shedding in *M. bovis* infected buffaloes, which may advance our knowledge of the epidemiology of bTB. Since oronasal swabs were stored in PrimeStore MTM, all microorganisms, including viral and bacterial pathogens, were inactivated, which would potentially permit movement of samples without risk of accidental pathogen spread. PrimeStore MTM stabilises DNA at room temperature, which also facilitates field collection and processing prior to performing the Ultra assay. Since the Ultra equipment is portable and can be easily operated without extensive training, the next step would be to determine if this is a field-friendly screening tool. The oronasal swab Ultra assay provides a more rapid ante-mortem test result than culture (which typically takes 56 days) to confirm the presence of MTBC. Based on the relatively large proportion of shedders that were detected in our cohort, future work should include serial monitoring of shedding in a group of *M. bovis* infected buffaloes to evaluate disease transmission risk under different conditions to improve management and understanding of the epidemiology of bTB in wildlife.

## Methods

### Animals and sample collection

A cohort of buffaloes that was part of the 2019 annual bTB test-and-slaughter program (n = 339) in the bTB-endemic Hluhluwe–iMfolozi Park (HiP), KwaZulu Natal, South Africa, were included in this study. Capture, immobilisation, sample collection, and bTB testing were performed as previously described^13,19^. Buffaloes were considered bTB test-positive if they had a positive result on any of the following tests: SCITT^20^, in vitro bovine Cattletype IGRA^21^, or the bovine IPRA^22^. Cytokine release assays used plasma harvested from whole blood stimulated with *M. bovis* specific antigens, ESAT-6 and CFP-10, in the QuantiFERON-TB Gold Plus tubes(QFT)^21,22^. The QFT IPRA and IGRA results were determined by quantifying the differential cytokine plasma concentrations between the QFT Nil and QFT TB antigen tubes, and assigning positive and negative outcomes, based on previously determined thresholds^19,22^. Test-positive buffaloes, and three randomly selected buffaloes that were negative on all three bTB tests, were culled and underwent post-mortem examinations, where various tissue samples (including tonsils, lung, abdominal serosa, retropharyngeal, mediastinal, and tracheobronchial lymph nodes) were collected and stored at −20 °C until further processing, as previously described^13,23,24^. In addition, post-mortem oropharyngeal swab samples were collected from the same culled buffaloes and stored in PrimeStore MTM (Longhorn Vaccines and Diagnostics, San Antonio, Texas, USA) at ambient temperature until further processing, as previously described^13^. Previously collected oropharyngeal swabs (n = 20) from immobilised buffaloes originating from historically bTB-free herds were also included as negative controls.

Ethical approval for this study was granted by Stellenbosch University Animal Care and Use Committee (ACU-2019-9081). Permission to perform animal research in terms of section 20 of the Animal Diseases Act was granted by the South African Department of Agriculture, Land Reform and Rural Development (DALRRD), formerly the Department of Agriculture, Forestry and Fisheries (DAFF), South Africa (12/11/1/7/2). All buffaloes were handled by the Ezemvelo KZN Wildlife veterinarians and game capture teams according to their guidelines. ARRIVE guidelines for reporting animal research have been followed as much as possible (https://arriveguidelines.org/).

### Xpert MTB/RIF ultra assay

The presence of MTBC DNA in oropharyngeal swab samples was determined using the Xpert MTB/RIF Ultra assay (Cepheid, Sunnyvale, California, USA), according to the manufacturer’s instructions, as previously described^13,15^. The Ultra assay results are based on the quantity of MTBC DNA that is detected in a sample, and reported as MTBC detected high, medium, low, very low, trace, or not detected. For this study, “MTBC not detected” was regarded as a negative result, whereas all the other detected ranks were considered a presumptive positive test result. The Ultra assay also reports a rifampicin resistance result for each sample, but this was not reported, since it was irrelevant in the context of this study.

### Mycobacterial culture and species identification

Tissue samples, including retropharyngeal, mediastinal, and tracheobronchial lymph nodes, lung, tonsils and abdominal serosa, underwent mycobacterial culture using the standard mycobacterial culture protocol with the BACTEC™ MGIT™ 960 Mycobacterial Detection System (Becton Dickinson, Franklin Lakes, New Jersey, USA) in a biosafety level 3 laboratory at Stellenbosch University, as previously described^24^. Culture-positive samples underwent PCR with primers targeting the genomic RDs 1, 4, 9 and 12 to confirm the presence of *M. bovis* in these samples^14^.

Oronasal swabs were not cultured, since they were stored in PrimeStore MTM, which inactivates all microorganisms, while stabilising nucleic acids, thereby improving human and animal safety during sample collection and transport.

## References

[1] Corner LAL, Murphy D, Gormley E (2011). *Mycobacterium bovis* infection in the Eurasian badger (*Meles meles*): the disease, pathogenesis, epidemiology and control. J. Comp. Pathol..

[2] de la Rua-Domenech R (2006). Ante mortem diagnosis of tuberculosis in cattle: A review of the tuberculin tests, γ-interferon assay and other ancillary diagnostic techniques. Res. Vet. Sci..

[3] Michel A (2006). Wildlife tuberculosis in South African conservation areas: implications and challenges. Vet. Microbiol..

[4] de Vos V (2001). The epidemiology of tuberculosis in free-ranging African buffalo (*Syncerus caffer*) in the Kruger national park, South Africa. Onderstepoort J. Vet. Res..

[5] Renwick AR, White PCL, Bengis RG (2007). Bovine tuberculosis in southern African wildlife: A multi-species host-pathogen system. Epidemiol. Infect..

[6] Walter WD (2012). On-farm mitigation of transmission of tuberculosis from white-tailed deer to cattle: Literature review and recommendations. Vet. Med. Int..

[7] Talip BA, Sleator RD, Lowery CJ, Dooley JSG, Snelling WJ (2013). An Update on global tuberculosis (TB). Infect. Dis. Res. Treat..

[8] Bernitz N (2021). Review of diagnostic tests for detection of *Mycobacterium bovis* infection in South African wildlife. Front. Vet. Sci..

[9] Schiller I (2010). Bovine Tuberculosis: A review of current and emerging diagnostic techniques in view of their relevance for disease control and eradication. Transbound. Emerg. Dis..

[10] Lorente-Leal V (2019). Validation of a real-time PCR for the detection of *Mycobacterium tuberculosis* complex members in bovine tissue samples. Front. Vet. Sci..

[11] Wood RC (2015). Detection of *Mycobacterium tuberculosis* DNA on the oral mucosa of tuberculosis patients. Sci. Rep..

[12] Wood RC (2021). Characterization of oral swab samples for diagnosis of pulmonary tuberculosis. PLoS ONE.

[13] Clarke C (2021). Novel molecular transport medium used in combination with Xpert MTB/RIF ultra provides rapid detection of *Mycobacterium bovis* in African buffaloes. Sci. Rep..

[14] Warren RM (2006). Differentiation of *Mycobacterium tuberculosis* complex by PCR amplification of genomic regions of difference. Int. J. Tubercul. Lung Dis..

[15] Goosen WJ (2020). The Xpert MTB / RIF Ultra assay detects *Mycobacterium tuberculosis* complex DNA in white rhinoceros (*Ceratotherium simum*) and African elephants (*Loxodonta africana*). Sci. Rep..

[16] Serrano M (2018). Different lesion distribution in calves orally or intratracheally challenged with *Mycobacterium bovis*: implications for diagnosis. Vet. Res..

[17] Santos N, Almeida V, Gortázar C, Correia-Neves M (2015). Patterns of *Mycobacterium tuberculosis*-complex excretion and characterization of super-shedders in naturally infected wild boar and red deer. Vet. Res..

[18] Moule MG, Cirillo JD (2020). *Mycobacterium tuberculosis* dissemination plays a critical role in pathogenesis. Front. Cell. Infect. Microbiol..

[19] Parsons SDC (2011). Modification of the QuantiFERON-TB Gold (In-Tube) assay for the diagnosis of *Mycobacterium bovis* infection in African buffaloes (*Syncerus caffer*). Vet. Immunol. Immunopathol..

[20] Smith K (2021). Optimisation of the tuberculin skin test for detection of *Mycobacterium bovis* in African buffaloes (*Syncerus caffer*). Prevent. Vet. Med..

[21] Bernitz N (2018). Detection of *Mycobacterium bovis* infection in African buffaloes (*Syncerus caffer*) using QuantiFERON®-TB Gold (QFT) tubes and the Qiagen cattletype® IFN-gamma ELISA. Vet. Immunol. Immunopathol..

[22] Goosen WJ, Cooper D, Miller MA, van Helden PD, Parsons SDC (2015). IP-10 is a sensitive biomarker of antigen recognition in whole-blood stimulation assays used for the diagnosis of *Mycobacterium bovis* infection in African buffaloes (*Syncerus caffer*). Clin. Vaccine Immunol..

[23] Bernitz N (2019). Impact of *Mycobacterium bovis*-induced pathology on interpretation of QuantiFERON®-TB Gold assay results in African buffaloes (*Syncerus caffer*). Vet. Immunol. Immunopathol..

[24] Goosen WJ (2014). Agreement between assays of cell-mediated immunity utilizing *Mycobacterium bovis*-specific antigens for the diagnosis of tuberculosis in African buffaloes (*Syncerus caffer*). Vet. Immunol. Immunopathol..

